# A scoping review of theories used to investigate clinician adherence to clinical practice guidelines

**DOI:** 10.1007/s11096-022-01490-9

**Published:** 2022-11-16

**Authors:** Derek Stewart, Moza Al Hail, Samaher Al-Shaibi, Tarteel Ali Hussain, Nada Nabil Abdelkader, Abdulrouf Pallivalapila, Binny Thomas, Wessam El Kassem, Yolande Hanssens, Zachariah Nazar

**Affiliations:** 1 College of Pharmacy, QU Health, Qatar University, Doha, Qatar; 2 Women’s Wellness and Research Center, Hamad Medical Corporation, Doha, Qatar; 3 Hamad Medical Corporation, Doha, Qatar

**Keywords:** Adherence to clinical guidelines, Clinical practice guidelines, Clinical pharmacy, Framework, Evidence-practice gap

## Abstract

**Background:**

Routine utilization of evidence-based clinical practice guidelines (CPGs) is an effective strategy to optimize patient care and reduce practice variation. Healthcare professionals’ failure to adhere to CPGs introduces risks to both patients and the sustainability of healthcare systems. The integration of theory to investigate adherence provides greater insight into the often complex reasons for suboptimal behaviors.

**Aim:**

To determine the coverage of literature surrounding the use of theory in studies of CPG adherence, report the key findings and identify the knowledge gaps.

**Method:**

In April 2021, three bibliographic databases were searched for studies published since January 2010, adopting theory to investigate health professionals’ adherence to CPGs. Two reviewers independently screened the articles for eligibility and charted the data. A narrative approach to synthesis was employed.

**Results:**

The review includes 12 articles. Studies were limited to primarily investigations of physicians, quantitative designs, single disease states and few countries. The use of behavioral theories facilitated pooling of data of barriers and facilitators of adherence. The domains and constructs of a number of the reported theories are captured within the Theoretical Domains Framework (TDF); the most common barriers aligned with the TDF domain of environmental context and resources, fewer studies reported facilitators.

**Conclusion:**

There is emerging use of behavioral theories investigating physicians’ adherence to CPGs. Although limited in number, these studies present specific insight into common barriers and facilitators, thus providing valuable evidence for refining existing and future implementation strategies. Similar investigations of other health professionals are warranted.

**Supplementary Information:**

The online version contains supplementary material available at 10.1007/s11096-022-01490-9.

## Impact Statements

- Investigations underpinned with behavioural theory provide more robust and generalizable evidence of healthcare professionals’ failure to adhere to Clinical Practice Guidelines, such data is essential to inform the refinement of existing and future implementation strategies.
- The domains and constructs of a number of the reported theories are captured within the Theoretical Domains Framework, thus demonstrating its versatility and advantage for such studies.
- Further investigations of physicians in alternative settings and of other health professionals are warranted to confirm the generalizability and the association of the existing literature.

## Introduction

Healthcare systems continuously seek strategies to enhance the quality of service delivery through promoting evidence-based practice and addressing inefficiencies. The implementation and routine utilization of clinical practice guidelines (CPGs) has long since been proposed to be an effective strategy to optimize patient care and reduce practice variation [1]. Equally, there is established consensus within the literature that failure to systematically develop, implement, and adhere to CPGs introduces risks to both patients and the sustainability of healthcare systems [1, 2].

For example, implementation investigations, which refers to the extent to which efficacious health interventions, such as CPGs, are effectively integrated into real-world clinical service systems [3]; have revealed the influence of numerous multilevel (patient, provider, team, organization system), often competing, factors that makes implementation of clinical guidelines complex and challenging[4–6]. Similarly adherence, which refers to the degree in which one’s behaviour coincides with recommendations [7], such as those included in CPGs, has been widely investigated, reporting a multitude of reasons for non-adherence to CPGs (e.g. patient preference, contra-indications, lack of knowledge) and its potential to result in suboptimal healthcare delivery and inefficiencies[1, 8–10].

The definition of CPGs most frequently cited is that of Field and Lohr, “systematically developed statements to assist practitioner and patient decisions about appropriate health care for specific clinical circumstances” [11]. Since the 1980s, there has been a rapid increase in the number and scope of CPGs; subsequent studies have reported their outcomes. Systematic reviews focusing on numerous clinical areas have reported improved patient health outcomes, reduced admission rates and length of hospital stay, less resource utilization and decreased medical costs [12–18]

However, the development of CPGs is complex and not without its challenges. Panel composition influencing recommendations; multiple scoring systems for the quality of evidence and ‘grades of recommendations’ and the limitations thereof; and timely guideline updating are but a few of the issues that warrant significant consideration in order to develop credible CPGs and necessitate appropriate critical appraisal skills of clinicians in order to advantage from them [19]. Furthermore, the rapid increase in the number of CPGs produced by different organizations on the same or similar topics, which can either agree or disagree with each other, can generate uncertainty in clinicians and patients about optimal recommendations, and instill doubt in the process of CPG development [20, 21]. Thus, for CPGs to benefit outcomes, specific attention is required to the processes of their dissemination, implementation and adherence. Indeed, there is an increasing body of literature reporting on the effectiveness of interventions to improve these processes and thereby enhance the routine use of CPGs in clinical settings. Relevant systematic reviews have focused on investigating interventions to uptake CPGs or best practices that target specific clinicians, including physicians [22–25], nurses [26–29] and allied health professionals [30–33]. These reviews have reported mixed outcomes, with only two conclusively reporting a positive improvement on professional outcomes such as knowledge and practice behaviors [31, 32].

Eccles et al. propose that adopting theory in such investigations provides further valuable insight on how determinants (e.g. physician attitude) influence the association between processes and outcomes, and facilitates more detailed understanding of strategies that may mitigate against determinants to support processes associated with desirable outcomes [34]. One subsequent scoping review sought to investigate how theory (including models and frameworks) had been employed to plan or evaluate the implementation and use of guidelines among physicians [35]. The review revealed that a range of theories (or models/frameworks) have been utilized in different aspects of individual study designs, and positive outcomes were achieved only in the few studies that had applied theory to evaluate interventions. The review also concluded that reported studies did not explicitly link pre-identified determinants of guideline use to specific theoretical constructs; and subsequently recommended further research to establish the number of types of theories that result in improved guideline use to help understand why theory-informed interventions fail to consistently achieve desired outcomes [35].

Similarly a systematic review by Davies et al. on studies evaluating guideline dissemination or implementation strategy targeting physicians concluded greater use of explicit theory, specifically behavioral change theories, is required to understand barriers, design interventions, and explore mediating pathways and moderators [36]. These theories intend to provide greater insight into the often complex reasons for suboptimal behaviors [37, 38]

Such recommendations are closely aligned to those of the United Kingdom (UK) Medical Research Council (MRC) guidance on ‘Developing and implementing complex interventions’, which attributes theory a central role within the process [37].

While reviews have focused on the stage of implementation of CPGs and the use of theory, none have reported the key process of adherence.

### Aim

The aims of this scoping review were to determine the coverage of literature surrounding the use of theory in studies of CPG adherence, report the key findings and identify the knowledge gaps.

## Method

The Preferred Reporting Items for Systematic Reviews and Meta-Analyses extension for Scoping Reviews (PRISMA-ScR) checklist guided the conduct and reporting of this review [39, 40].

### Inclusion criteria

Studies reporting any health professional practicing in any setting which applied any theory or theoretical framework to study adherence to clinical guidelines were included in the review; this included both interventional and non-interventional studies. Studies which focused on implementation were excluded. The search included peer-reviewed studies published in English from January 2010 until April 2021 were included; editorials, commentaries, abstracts and letters were excluded.

### Search strategy

The search was conducted in PubMed, Cumulative Index to Nursing and Allied Health Literature (CINAHL) and Scopus. References lists of included articles were reviewed for inclusion. The following Medical Subject Headings [MeSH] and keywords were adapted to each database using ‘OR’: "theor*", "framework(s)". These were combined with "guideline(s) using ‘AND’. The results from this search were combined with the following using ‘AND’: "health personnel", "clinician(s)", “delivery of health care", "practitioner(s)".

All articles were exported to the support platform for the development of systematic reviews Rayyan QCRI® [41], and duplicates were removed. Two reviewers independently screened titles and abstracts followed by full text for eligibility, with disagreements resolved by discussion or consultation with a third reviewer.

### Data extraction

Data were independently extracted by two reviewers using a standardized pilot data collection tool. The following data were extracted: study aim; setting, study design; simple size; health professionals studied; and the theory or theoretical framework used.

### Synthesis

A narrative approach to data synthesis was employed to pool the evidence on how the theories and theoretical frameworks were used, and the main findings in relation to the aim around clinical guideline adherence. These findings were mapped to key behavioural determinants of adherence.

## Results

**Fig. 1:**
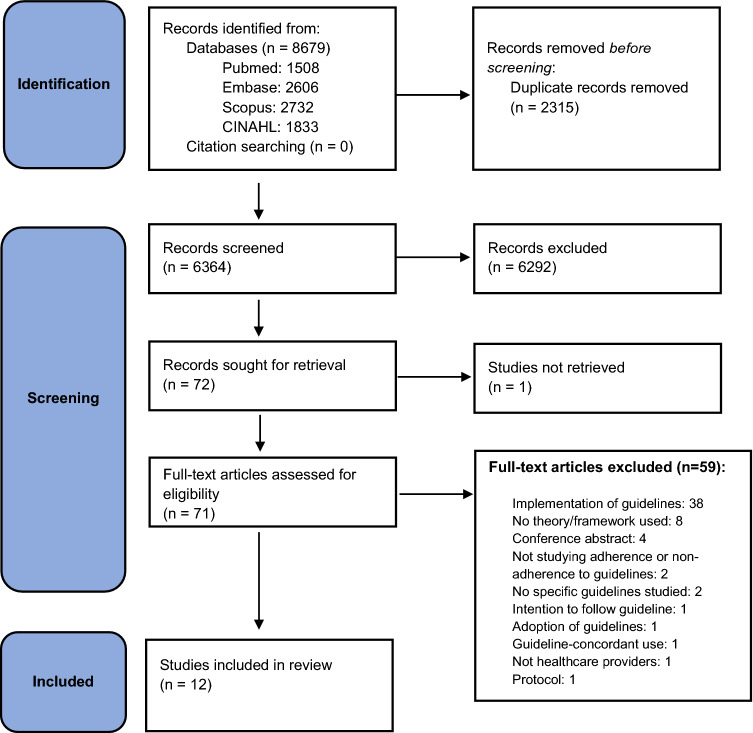
PRISMA flow diagram of the study selection process

A total of 8679 studies were identified from the search, with 6364 remaining following removal of duplicates. Review of titles and abstracts resulted in 71 full-text studies being screened and 12 retained for data extraction and synthesis (Table 1). The main reason for exclusion at full-text review was the study outcomes of implementation and not adherence (*n* = 38) (see Fig. 1).

**Table 1:** Study Characteristics

Author name, Publication year, Country	Aims/ Objective	Setting	Study design	Sample size, Target healthcare professionals
Backman et al., 2015, United Kingdom (UK)[53]	Explore barriers to, and enablers, of adherence to current guidance for the diagnosis and management of suspected encephalitis, matching these to behavior change techniques	Two specialist hospitals and four teaching hospitals, UK	Qualitative design, one-to-one semi-structured interviews and one focus group	43 professionals (interviews with 5 consultants, 28 junior doctors, 7 nurses and health care assistants; one focus group of 5 consultants)
Bussières et al., 2012, North America (USA and Canada) [51]	Explore beliefs about adherence to evidence-based diagnostic imaging guideline recommendations for spinal disorders	Multi-centered: American Specialty Health Network service (California & Georgia) and professional associations (Ontario & Quebec, Canada)	Qualitative design, focus groups	21 licensed chiropractors in 6 focus groups
Cartoos et al., 2012. Belgium [50]	Explore factors affecting antimicrobial guideline use and how this could be improved	1900 bed, university teaching hospital, Belgium	Quantitative design, cross-sectional survey (prior focus groups to develop questionnaire content)	195 physicians responded (50.5% corrected response rate)
Efstathiou et al., 2011, Cyprus[52]	Explore factors that influence nurses’ compliance with guidelines to prevent exposure to microorganisms	Two public hospitals, Cyprus	Qualitative design, focus groups	30 nurses in 4 focus groups
Glauser et al., 2012, USA [42]	Better understand barriers and perceptions underlying adherence to recommendations from the cystic fibrosis guidelines	Cystic fibrosis centers, USA	Quantitative design, cross-sectional survey	133 pediatric pulmonologists representing 92 centers (78.2% response rate)
Lugtenberg et al., 2011, Netherlands [48]	Assess perceived barriers in adhering to guideline recommendations (eye inflammation, cerebrovascular accident, urinary tract infection, thyroid disorders)	All General Practitioners (GPs) in Southwestern Netherlands	Quantitative design, cross-sectional survey (prior focus groups to develop questionnaire content)	264 GPs (38% response rate)
De Ruijter et al., 2017, Netherlands[49]	Explore socio-cognitive determinants of nurse adherence to smoking cessation guidelines	General practices	Qualitative design, one-to-one semi-structured interviews	19 practice nurses
Radwan et al., 2017, Palestine [46]	Explore influence of organizational culture on adherence to clinical practice guidelines for diabetes mellitus	71 primary health care centers, Palestine	Quantitative design, cross-sectional survey, interviewer administered questionnaire	225 family doctors and 98 nurses (93.3% response rate)
Radwan et al., 2017, Palestine [45]	Explore perceived barriers to adherence to clinical practice guidelines for diabetes mellitus	71 primary health care centers, Palestine	Quantitative design, cross-sectional survey, interviewer administered questionnaire	225 family doctors and 98 nurses (93.3% response rate)
Radwan et al., 2018, Palestine[47]	Explore perspectives and experiences towards the main barriers influencing adherence to diabetes guidelines	Palestinian Primary Health Care Ministry of Health and Primary Health Care United Nations Relief and Work Agency	Qualitative design, semi-structured interviews	16 senior doctors and 4 nurses
Waddimba et al., 2016. USA [43]	Examine the indirect, moderating effect of physicians’ satisfaction with their practice on their adherence to specific diabetes clinical guidelines	Independent Practice Association (not-for-profit physician organization), USA	Quantitative design, cross-sectional survey	156 internists and family practitioners (no response rate provided, specific population meeting criteria)
Zhu et al., 2018, USA [44]	Identify Primary Care Providers’(PCPs) adherence and associated barriers to American Academy of Pediatrics dental referral standards for children less than 4 years	PCPs for Medicaid enrolled children, USA	Quantitative design, cross-sectional, vignette-based survey	219 physicians (50.3% response rate)

### Study characteristics

Table 2 gives the study characteristics. Studies were conducted in the United States (US) [42–44], Palestine [45–47], the Netherlands [48, 49], Belgium [50], Canada/US [51], Cyprus [52] and the United Kingdom [53]. The most common methodology was a cross-sectional survey [42–46, 48, 50], with the questionnaires used in two studies being informed by prior qualitative research [48, 50]. Survey participant numbers ranged from 133 to 264 [42, 48], with response rates of 38.0% to 93.3% [45, 46, 48]. Participants were largely physicians, with a smaller number of nurses, representing hospitals and family medicine facilities. Five qualitative studies were reported, two of which employed focus groups [51, 52], two semi-structured interviews [47, 49], and one a combination of focus groups and semi-structured interviews [53]. A total of 126 individuals participated in the qualitative studies, ranging from 16 to 43 comprising physicians, nurses, chiropractors and health care assistants, representing hospitals, family medicine facilities and research centres.

**Table 2:** Theories/Frameworks used and Study Findings

Author name, Publication year, Country	Theory or framework used	How theory was used	Main findings in relation to the aim and the theory used
Backman et al., 2015, UK [53]	Theoretical Domains Framework (TDF)	Data generation (interview schedule/focus group topic guide based on TDF) Data analysis, coding Mapped domain findings to defined behavior change techniques	Barriers to guidance adherence aligned to TDF domains throughout the patient journey. Key issues around clinical assessment and investigation. Assessment influenced by clinical environment and poor decision-making processes (knowledge, memory, attention, and decision-making, environmental resources, context). Professionals lacked confidence in performing procedures (beliefs about capabilities) and challenges of busy clinical schedule (environmental context and resources). Concerns expressed regarding over-treatment, when to stop and failure to confirm diagnosis (beliefs of consequences, professional role). Intervention developed by mapping behavior change techniques to TDF domains
Bussières et al., 2012, USA and Canada [51]	TDF	Data generation (focus group topic guide based on TDF) Data analysis, coding	Most significant factors were beliefs about consequences (centered on ordering x-rays, attitudes about guidelines), professional role and identity (autonomy, norms), beliefs about capabilities (with unclear diagnosis), social influences (colleagues, formal training, publications) and knowledge (guideline awareness, agreements)
Cartoos et al., 2012. Belgium [50]	Theory of Planned Behavior (TPB) (supplemented with Self-Report Index of Habit Strength)	Data collection (questionnaire items aligned to TPB domains, with additional items from Self-Report Index of Habit Strength and focus group findings)	The overall intention toward using guidelines not very predictable. Perceived behavioural control significant predictor. Determinants different for residents and faculty; faculty’s intentions significantly influenced by habit and residents by perceived behavioral control
Efstathiou et al., 2011, Cyprus [52]	Health Belief Model (HBM)	Data generation (focus group topic guide informed by HBM) Data analysis, coding	Factors contributing to non-compliance applied to main domains of HBM: barriers (e.g. availability of equipment, patients’ discomfort, too busy, lack of nurses, implementation time consuming, psychological factors, physicians’ influence); susceptibility (risk of infection); benefits (protection from infection, psychological factors); cues to action (previous exposure, reminding, patients’ personal characteristics); severity (fear, serious negative impact, costs from infection)
Glauser et al., 2012, USA [42]	Cabana Framework	Data collection (questionnaire items aligned to Cabana Framework domains)	Pulmonologists adhered to a high degree. Almost half endorsed patient barriers (high cost, burdensome regimens, non-adherence) as preventing guideline adherence. Higher outcome expectancy and fewer environmental/system barriers significantly associated with adherence. Trend for association between familiarity and adherence evident
Lugtenberg et al., 2011, Netherlands[48]	Cabana Framework	Data collection (barriers identified in focus groups were classified in accordance to Cabana framework and used in constructing questionnaire items)	Mean perceived adherence rate across all guidelines relatively high. Barriers related to external factors (patient ability, behavior, preferences). Barriers related to lack of applicability of guideline. Adherence negatively associated with overall perceived barriers
De Ruijter et al., 2017, Netherlands [49]	I-Change Model and the Diffusion of Innovations Theory	Data generation (interview guide developed based on I-change model and Diffusion of Innovations Theory)	Barriers contributing to poor adherence largely psychological and practical. Psychological included low self-efficacy to motivate patients and arranging adequate follow-up consultations. Practical barriers included outdated guidelines, and lack of high-quality trainings
Radwan et al., 2017, Palestine [46]	Competing Values Framework (CVF)	Data collection (CVF questionnaire was translated to Arabic)	Guideline adherence moderate. Clan/group culture most prevalent in the Palestinian Primary Healthcare Centers (PHC) of the Ministry of Health (MoH). Hierarchical culture most prevalent in the PHC- United Nations Relief and Works Agency for Palestine Refugees (UNRWA)
Radwan et al., 2017, Palestine [45]	Cabana Framework	Data collection (questionnaire items aligned to Cabana Framework domains)	Overall adherence moderate. Key barriers in MoH were behavioral; lack of resources, lack of incentives, lack of trustworthiness in guidelines. In UNRWA, lack of time, lack of trustworthiness in guidelines most common barriers to adherence
Radwan et al., 2018, Palestine [47]	Cabana Framework	Data generation (interview guide based on Cabana Framework categories) Data analysis (coding)	Environmental factors most major barrier, particularly lack of incentive, lack of resources. Further barriers were guideline trustworthiness and outdated recommendations
Waddimba et al., 2016. US [43]	Theory of Planned Behavior and Theory of Work Adjustment (TWA)	Data collection (questionnaire items aligned to TPB and TWA)	Physician satisfaction had significant indirect effect on performance. Those discontented with practice only motivated to adhere to guidelines that aligned with personal attitudes. Discontented physicians less likely to adhere to guidelines when sensing social pressure to comply. For satisfied physicians, neither personal attitudes, subjective norms, nor perceived behavioral control significantly affected behavior
Zhu et al., 2018, USA [44]	Cabana Framework	Data collection (questionnaire items aligned to Cabana Framework)	Moderate guideline adherence. Inadequate workforce reduced adherence for most levels. Adherence linked to proper knowledge of risk status, risk assessment barriers, and pediatric practice. Knowledge of dental caries risk and doctor specialty strongest and most consistent predictive factors of adherence

### Study aims

Study aims focused on issues of adherence and associated beliefs, and perceptions of barriers and facilitators relating to an array of clinical guidelines. The guidelines largely focused on single disease states or therapeutics issues, specifically diabetes mellitus in four studies [43, 45–47], and one study each for antimicrobials [50], cystic fibrosis [42], dental referral [44], exposure to microorganisms [52], smoking cessation [49], spine disorders [51] and suspected encephalitis [53]. One study targeted four different guidelines (red eye, cerebrovascular accident, urinary tract infection and thyroid disorders) [48].

### Theories and theoretical frameworks used

Table 2 summarizes the theories and theoretical frameworks used, the rationale for use provided by the authors, how they were used, and the main findings. All are related to aspects of behavior and behavior change. The Cabana Framework was the most frequently used [42, 44, 45, 47, 48], followed by the Theoretical Domains Framework (TDF) [51, 53], the Theory of Planned Behavior (TPB) [50], Competing Value Framework [46], and the Health Belief Model (HBM) [52]. Two studies utilized a combination of theories; I-Change Model and the Diffusion of Innovations Theory [49], TPB and Theory of Work Adjustment (TWA) [43].

The domains or constructs of the theories and theoretical frameworks are illustrated in Supplementary File 1.

Theories and frameworks were largely justified in terms of the underlying domains and constructs in relation to the study aims. Other reasons included the tradition of using a particular theory or framework in studying particular behaviors or in specific clinical settings. For example, Backman et al. reported that TDF had been applied in a variety of settings and studies of clinical behaviors [53]. Cartoos et al. noted that the Theory of Planned Behavior had been used effectively in the evaluation of medical practice, including antibiotic use [50]. No justification was provided in two studies [44, 48], both of which used the Cabana Framework. Questionnaires were derived with reference to the theories and theoretical frameworks in the cross-sectional surveys; these aided the development of data generation tools and analytical coding frameworks in the qualitative studies.

### Main findings in relation to the aims

The use of behavioral theories and theoretical frameworks facilitated pooling of data of barriers and facilitators of adherence. In addition, the domains and constructs of a number of the behavioral theories (e.g., TPB, HBM) are captured within TDF. Almost all studies reported barriers to adherence, the most common aligning with the TDF domain of environmental context and resources. Participants reported several related factors limiting adherence, including busy schedules [42, 44, 45, 47, 52, 53], guidelines being outdated or a perceived lack of trustworthiness in the recommendations [45, 47, 49], practising according to the guidelines considered burdensome [42, 52], lack of specific resources [45, 47, 53] and insufficient training [49]. There were also issues relating to knowledge of the existence of guidelines and their content [42, 44, 48, 51, 53]. Several studies reported barriers aligning to the belief of consequences domain. There were reports of concern that adhering to the guidelines would deliver the clinical gains claimed [1, 2, 4, 11]. There were also issues of belief of capabilities in being able to apply the guideline recommendations [49][1, 2, 4, 7]. TDF domains which emerged less commonly were those relating to social influences, including other health professionals not adhering [4] and pressure from patients not to adhere [6]. Two studies reported the issues of behavioral regulation, notably the lack of incentives to adhere [9, 10]. Memory, attention and decision making was noted to be an issue in one study, specifically remembering to apply the guideline [1].

Fewer studies reported facilitators to guideline adherence. These included belief of consequences of the clinical gain to be achieved [4] and cost savings [5]. Participants in one study cited guideline adherence to be part of their professional role and identity [12], while others noted constant reminders as a positive influence [4].

## Discussion

The key finding of this scoping review is that a limited number of studies have applied any theory to explore health professionals’ adherence to CPGs. Use of TDF (or individual behavioural theories integral to TDF) identified barriers to adherence relating to the environmental context and resources, beliefs of consequences, beliefs of capabilities and aspects of knowledge. Few studies reported any facilitators to adherence.

This scoping review was conducted according to best practice through the application of rigorous and transparent processes [39, 40]. Munn et al. suggest that scoping reviews are particularly appropriate for reporting the coverage of literature and examining evidence when it is uncertain if more specific questions can be answered through conducting a systematic review [54]. One further key difference compared to a systematic review is the absence of quality assessment of included studies. Limitations of the review are the index search date of 2010 and restricting the search to CPGs. It is likely that extending the review to other forms of guidelines would have captured a greater body of work. Furthermore, it is conceivable that the related concepts of *adoption* and *implementation* are reported on in the literature without clear distinction; further complexity may be added by the variation in terminology and classification of terms across countries. Indeed, a review of research funding agencies in nine countries revealed 29 distinct terms referring to aspects of dissemination and implementation research [55].

This scoping review has identified a paucity of theory informed studies, with only 12 identified from seven countries, which contrasts with the vast number of systematic and scoping reviews on the wider literature of health professionals’ adherence to CPGs. There is therefore a clear gap in the literature in terms of the number and coverage of studies. This gap is reinforced given that most studies were cross-sectional surveys with less qualitative studies and no mixed methods studies. The lack of studies must be borne in mind when interpreting the findings.

Theories were largely used to develop data collection tools and analytical frameworks, with the most common being the Cabana Framework and TDF. TDF is an integrative framework of 33 behaviour change theories and 128 theoretical constructs, described in 14 overarching domains [56, 57]. Of note, three further studies used the HBM, TPB and Diffusion of Innovations, which are included in the 33 theories captured in TDF. While there is some similarity between the theories identified in the scoping review of implementation studies by Liang et al. [35], it must be acknowledged that implementation and adherence are very different, albeit related processes. Implementation studies are more likely to apply implementation theories and frameworks, such as the Consolidated Framework for Implementation Research (CFIR) [58], rather than adherence studies which are more likely to focus on behavior change theories and frameworks such as TDF.

There are a number of benefits to applying theory, including enhancing the robustness and rigour, and the relevance and impact of research findings. Theories provide comprehensive explanations, e.g., the 14 domains TDF represent the determinants of (influences on) any behaviour which may be facilitators, barriers, or have no or little influence. Using theory enables researchers to connect pieces of research data to generate findings which fit into a collation of other studies [59]. The robust and rigorous evidence of behavioural determinants can aid the development of targeted interventions, which are more likely to be effective and sustained rather than those developed more pragmatically. The UK Medical Research Council guidance, ‘Developing and implementing complex interventions’ [60], emphasizes the importance of theory in the development stage. ‘Complex interventions’ are essential those with multiple interacting components and players, which aligns to the processes and number of potentially influences on CPG adherence.

Key barriers to adherence were TDF domains of environmental context and resources (e.g., outdated guidelines, burdensome guidelines, lack of resources), belief of consequences (e.g., adhering would not deliver clinical gains claimed), belief of capabilities (e.g., ability to apply CPGs) and knowledge (e.g., existence of CPGs). Those studies which used the Cabana Framework identified similar issues. These barriers can act as behaviour change intervention (BCI) targets, defined as `coordinated sets of activities designed to change specified behaviour patterns'. BCIs consist of interacting components known as `behaviour change techniques' (BCTs) which are `observable and replicable components designed to change behaviour' [61, 62]. Evidence based BCTs are mapped to specific TDF domains to facilitate intervention development [56, 57]. For example, BCTs mapped to beliefs of consequences include: anticipated regret (inducing or raising awareness of expectations of future regret); and comparative imaging of future outcomes (prompt or advise the imagining and comparing of future outcomes). These BCTs would form the basis of interventions which would be tested through the stages of feasibility and pilot testing, evaluation and implementation in future studies. There may be merit in conducting a systematic review to systematically review, critically appraise and synthesize the evidence on the application and use of theory in the development and evaluation of behaviour change interventions designed to improve health professionals’ adherence to CPGs. The Theory Coding Scheme (TCS) would aid assessment of the specific ways in which theory had been applied. Consisting of 19 items, provides a detailed and comprehensive checklist for assessing the extent to which behaviour change interventions are theoretically based [63]. One potential limitation to this systematic review would be the likely small number of relevant studies.

## Conclusion

There is emerging use of behavioural theories investigating physicians’ adherence to CPGs; notably the domains and constructs of a number of the reported theories are captured within the TDF. Although limited in number, these studies present specific insight into the barriers, which align to the *environmental context and resources* TDF domain, and less frequently the facilitators of physicians’ adherence to CPGs. This data provide valuable evidence for refining existing and the development of future implementation strategies. Similar investigations of physicians in alternative settings and of other health professionals are warranted to confirm the generalizability and the association of these findings.

## Supplementary Information

Below is the link to the electronic supplementary material.

Supplementary file1 (PDF 39 kb)
