# Supplementary material for: A scoping review of theories used to investigate clinician adherence to clinical practice guidelines
Source: Int J Clin Pharm. 2022 Nov 16;45(1):52–63. doi: 10.1007/s11096-022-01490-9 (PMC9938823; doi:10.1007/s11096-022-01490-9)
Supplement: Supplementary file 1 — Supplementary file1 (PDF 39 kb) [file 11096_2022_1490_MOESM1_ESM.pdf]

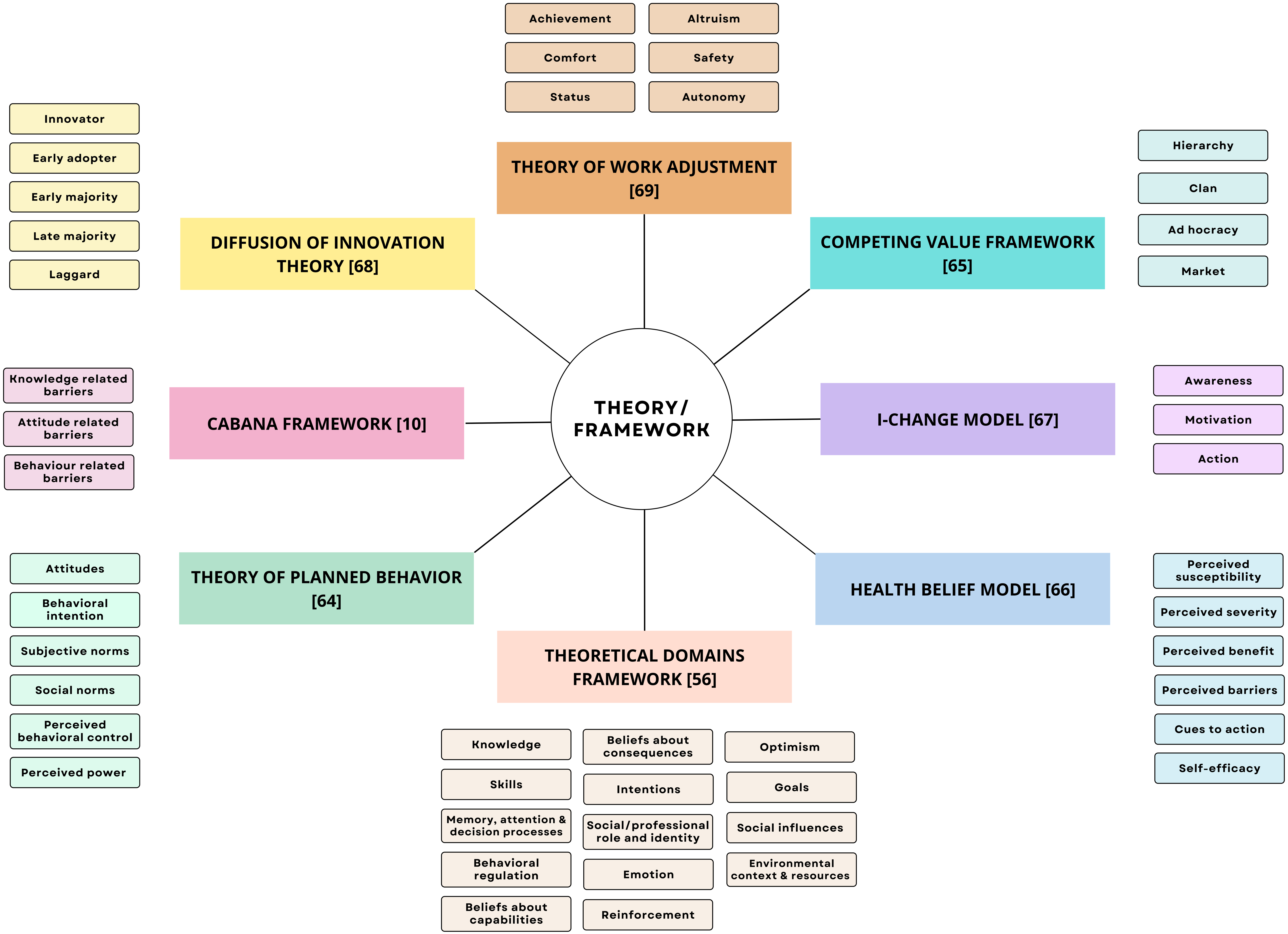

Supplementary File 1: The domains or constructs of the theories and theoretical frameworks adopted in the included studies.
